# Lung cancer risk prediction using augmented machine learning pipelines with explainable AI

**DOI:** 10.3389/frai.2025.1602775

**Published:** 2025-09-03

**Authors:** Pavithran M S, Saranyaraj D, Anirban Chakrabortty

**Affiliations:** School of Computer Science and Engineering, Vellore Institute of Technology, Chennai Campus, Tamil Nadu, India

**Keywords:** lung cancer prediction, class imbalance, explainable AI, lime, SMOTE

## Abstract

Lung cancer remains the leading cause of cancer-related deaths worldwide, making early and precise diagnosis is critical for improving the patient survival rates. Machine learning has shown promising results in predictive analysis for lung cancer prediction. However, class imbalance in clinical datasets negatively impacts the performance of Machine Learning classifiers, leading to biased predictions and reduced accuracy. In an attempt to address this issue, various data augmentation techniques were applied alongside classification models to enhance predictive performance. This study evaluates data augmentation techniques paired with machine learning classifiers to address class imbalance in a small lung cancer dataset. A comparative analysis was conducted to assess the impact of different augmentation techniques with classification models. Experimental findings demonstrate that K-Means SMOTE, combined with a Multi-Layer Perceptron classifier, achieves the highest accuracy of 93.55% and an AUC-ROC score of 96.76%, surpassing other augmentation-classifier combinations. These results underscore the importance of selecting optimal augmentation methods to improve classification performance. Furthermore, to ensure model interpretability and transparency in medical decision-making, LIME is utilized to provide insights into model predictions. The study highlights the significance of advanced augmentation techniques in addressing data imbalance, ultimately enhancing lung cancer risk prediction through machine learning. The findings contribute to the growing field of AI-driven healthcare by emphasizing the necessity of selecting effective augmentation-classifier pairs to develop more accurate and reliable diagnostic models. Due to the dataset’s high cancer prevalence (87.45%) and limited size, this work is a preliminary methodological comparison, not a clinical tool. Findings emphasize the importance of augmentation for imbalanced data and lay the groundwork for future validation with larger, representative datasets.

## 1 Introduction

Lung cancer is one of the most prevalent causes of cancer deaths worldwide, accounting for approximately 1.8 million deaths annually. Irrespective of the advances in medicine and treatment procedures, early detection is a significant concern. The five-year survival rate of lung cancer is significantly high in the initial stage of detection, but most of the cases are diagnosed in late stages due to the reason that the disease does not show any apparent sign in the initial stage. Thus, advanced and accurate prediction of lung cancer risk is vital to improve the outcome of patients and reduce mortality.

Conventional diagnosis techniques of lung cancer, including biopsy, imaging modalities (CT scans, PET scans, and X-rays), and molecular screening, are very effective yet costly, invasive, and require specialized medical skills. Further, most of the people at risk of developing lung cancer are not screened periodically, resulting in delayed diagnosis and fewer treatment options. This lacuna requires the building of new methods, particularly machine learning-based prediction models that can understand patient history, behavior, symptoms, and other risk factors and provide an early warning system for the identification of lung cancer. When learned over large datasets, machine learning models are capable of capturing latent patterns among patient data and giving risk scores for lung cancer. But one major problem with medical datasets is class imbalance, where cancer-positive instances are outnumbered significantly by non-cancerous instances. The class imbalance typically results in models biased toward the majority class, and thus low sensitivity and recall rates, so most of the cancer-positive instances go undetected. Data augmentation methods are used to solve this problem by creating synthetic minority samples, hence enhancing the model performance.

There has been a lot of research on applying machine learning and deep learning for the diagnosis of lung cancer. The major emphasis has been placed on image-based methods, including CNNs for CT scan analysis, and tabular model-based approaches involving patient demographics, symptoms, and lifestyle variables. For example, deep learning-based radiomics techniques have shown promise in the detection of lung cancer by image segmentation and feature extraction. These methods, however, require substantial, high-quality labeled datasets, which are often scarce in real-world clinical environments. Moreover, despite their accuracy, deep learning models lack interpretability, making it challenging for healthcare professionals to rely on their decisions. In contrast, machine learning-based classification models have been effectively applied to structured patient data. These models evaluate information such as smoking history, genetic predisposition, occupational exposure, and existing symptoms to determine lung cancer risk. Nevertheless, as previously noted, class imbalance remains a significant obstacle in achieving reliable predictions.

This work fills that gap by systematically evaluating multiple data augmentation techniques paired with various machine learning classifiers, providing a comparative analysis of their effectiveness in predicting lung cancer risk. Additionally, LIME is used to ensure model transparency and explainability. This study is driven by three key research questions: (1) How do different data augmentation techniques impact the performance of machine learning classifiers in lung cancer risk prediction? (2) Which augmentation-classifier combinations yield the most accurate and balanced predictions? (3) Can explainable AI methods like LIME provide transparent insights into prediction rationale across models? These questions frame the core of our study, which aims to contribute to the development of accurate and interpretable machine learning models for early lung cancer risk assessment.

This paper is organized as follows: Section 2 summarizes the existing research relevant to this work. Section 3 describes the dataset and the methodology employed in this work. Section 4 reports and discusses the experimental results. The conclusions are finally summarized in Section 5.

## 2 Literature survey

The evolution of machine learning and deep learning has largely played a crucial role in the early detection and diagnosis of illnesses, especially lung cancer. Research papers on different methodologies such as machine learning classifiers, deep learning architectures, data enhancement methods, and explainable AI models are discussed in this literature review. The hybrid models, quantum computing methods, and attention mechanisms have been identified to improve predictive accuracy in studies. Moreover, synthetic data generation, multi-omics integration, and risk prediction models research reveal cutting-edge solutions for medical diagnostics. This review highlights upcoming trends and challenges of AI-based healthcare applications with a focus on precision and efficiency. Maurya et al. (2024) have explored the results of various classification models by balancing the classes with the help of SMOTE. In our proposed research, various augmentation techniques combined with classification models with their justification were used to find the best strategy for lung cancer risk prediction. Limited attention has been paid to how augmentation methods interact differently with various classifiers in terms of predictive performance. This gap forms the basis of our research, which systematically evaluates multiple augmentation-classifier combinations and integrates interpretability using LIME to ensure transparency in medical decision-making. Table 1 presents the summary of existing research pertaining to this research.

**Table 1:** Summary of previous research on lung cancer prediction.

References	Title	Findings	Inference
Maurya et al. (2024)	Performance of Machine Learning Algorithms for Lung Cancer Prediction: A Comparative Approach	The most effective techniques for forecasting lung cancer in the early stages were Bernoulli Naive Bayes and K-Nearest Neighbor.	Machine learning algorithms can contribute substantially to early detection of lung cancer based on clinical information.
Sathe et al. (2024)	End-to-End Fully Automated Lung Cancer Screening System	Designed an automated system which has 92.09% segmentation accuracy, 94.18% volume estimation accuracy, and 96.4% grading accuracy.	AI-based automation enhances efficiency and accuracy in lung cancer screening.
Pellicer et al. (2023)	Data Augmentation Techniques in Natural Language Processing	Different augmentation methods were tested; back-translation was found useful.	Data augmentation enhances the generalization capability of models in NLP but needs to be better suited for NLP tasks.
Sinjanka et al. (2024)	ML-Based Early Detection of Lung Cancer: An Integrated and In-Depth Analytical Framework	Random Forest obtained 97.9% accuracy in early detection.	Machine learning has the potential to facilitate early detection of lung cancer, and hence timely intervention.
Kanber et al. (2024)	LightGBM: A Leading Force in Breast Cancer Diagnosis Through Machine Learning and Image Processing	LightGBM classified breast cancer with more than 99% accuracy.	LightGBM performs very well for cancer classification and can be utilized for lung cancer.
Chen et al. (2023)	Lung Cancer Prediction Using Electronic Claims Records: A Transformer-Based Approach	Transformer model obtained 0.668 AUC for all-stage lung cancer prediction.	Transformers are useful for lung cancer prediction with electronic health records.
Mohamed and Ezugwu (2024)	Enhancing Lung Cancer Classification and Prediction With Deep Learning and Multi-Omics Data	Achieved a 97% accuracy by considering mRNA, miRNA, and DNA methylation data.	Multi-omics data improves lung cancer classification accuracy.
Li et al. (2022)	A Novel Deep Learning Framework Based Mask-Guided Attention Mechanism for Distant Metastasis Prediction of Lung Cancer	Achieved an AUC of 0.822 on a mask-guided attention network.	Deep learning enhances metastasis prediction by considering tumor and lung regions.
Alsinglawi et al. (2022)	An Explainable Machine Learning Framework for Lung Cancer Hospital Length of Stay Prediction	Random Forest with SMOTE achieved 98% AUC for hospital stay prediction.	Explainable AI assists in interpreting patient hospital stay predictions.
Kesiku and Garcia-Zapirain (2024)	AI-Enhanced Lung Cancer Prediction: A Hybrid Model’s Precision Triumph	Lung cancer from clinical notes obtained 98.1% with a CNN-Bi-LSTM hybrid model	Hybrid deep learning models enhance text-based lung cancer detection.
Khalsan et al. (2022)	A Survey of Machine Learning Approaches Applied to Gene Expression Analysis for Cancer Prediction	Machine learning method was discussed over gene expression-based cancer prediction	Gene expression analysis with ML enhances biomarker discovery and cancer prediction.
Khanna et al. (2025)	Volatile Organic Compounds for the Prediction of Lung Cancer Using Ensemble Machine Learning	Proposed Ensemble models achieved a 100% accuracy for prediction of breath VOCs	VOCs are potential biomarkers for non-invasive lung cancer detection.
Ravindran and Gunavathi (2024)	Cancer Disease Prediction Using Integrated Smart Data Augmentation and Capsule Neural Network	Achieved over 98% by smart data augmentation and CapsNet in classification tasks.	Deep learning and data augmentation improve cancer prediction models.
Mohanty et al. (2025)	A Quantum Approach to Synthetic Minority Oversampling Technique	Proposed Quantum-SMOTE using quantum computing for data augmentation, improving class balance.	Quantum methods enhance traditional SMOTE techniques, making oversampling more effective in imbalanced datasets.
Amin et al. (2024)	Multimodal Non-Small Cell Lung Cancer Classification Using Convolutional Neural Networks	CNN-based multi-omics model attained high classification accuracy for NSCLC subtypes.	Multi-omics deep learning improves NSCLC subtype classification and treatment.
Javed et al. (2024)	Deep Learning for Lung Cancer Detection: A Review	Deep learning methods were discussed, with CNN having the highest accuracy.	CNN-based deep learning is still the best method for lung cancer detection.
Ahmed et al. (2024)	A Comparative Analysis of LIME and SHAP Interpreters With Explainable ML-Based Diabetes Predictions	Compared SHAP and LIME in interpreting ML models for diabetic prediction with 86% accuracy.	Explainable AI methods enhance model interpretability and can be applied to cancer predictions.
Modak et al. (2024)	GPD-Nodule: A Lightweight Lung Nodule Detection and Segmentation Framework	Proposed a lightweight framework using super-pixel generation for accurate lung nodule detection.	Effective lung nodule detection minimizes misdiagnosis and enhances early cancer detection.
Flyckt et al. (2024)	Pulmonologists-Level Lung Cancer Detection Using an Explainable ML Approach	ML model using routine blood tests and smoking history outperformed pulmonologists in LC detection.	ML models based on blood tests can facilitate early lung cancer detection.
Shaheen et al. (2025)	New AI Explained and Validated Deep Learning Approaches	Introduced LeDNet and HiDenNet deep learning models, achieving up to 86% accuracy for diabetes prediction.	Explainable AI improves model interpretability and enhances decision-making in medical diagnosis.
Wei et al. (2024)	ML for Early Discrimination Between Lung Cancer and Benign Nodules	Logistic Regression and XGBoost models achieved AUC of 0.716 and 0.913, respectively.	ML-based risk models increase early screening and staging accuracy for lung cancer.
Meeradevi et al. (2025)	Lung Cancer Detection with Machine Learning Classifiers and Deep Learning Model	Inception v3 deep learning model achieved 97.05% accuracy in classifying lung diseases.	Deep learning models outperform traditional ML classifiers for lung cancer detection.
Nazir et al. (2023)	ML-Based Lung Cancer Detection Using Multiview Image Registration and Fusion	Integrated multiple image views for improved cancer classification accuracy.	Multiview image fusion improves ML-based lung cancer detection.
Rao and Arshad (2023)	Early Detection of Lung Cancer Using ML Technique	Deep learning methods improved early-stage lung cancer detection.	AI-based methods help in efficient and precise early lung cancer diagnosis.
Thakur et al. (2023)	RNN-CNN Based Cancer Prediction Model for Gene Expression	Hybrid RNN-CNN model improved classification of gene expression for multiple cancer types.	Deep learning improves accuracy in cancer classification through gene expression data.
Murthy and Thippeswamy (2025)	TPOT with SVM Hybrid Model for Lung Cancer Classification	TPOT-SVM hybrid achieved 91.77% accuracy for lung cancer classification using CT images.	Automated ML pipeline optimization enhances lung cancer classification performance.
Alzahrani (2025)	Early Detection of Lung Cancer Using Predictive Modeling Incorporating CTGAN Features and Tree-Based Learning	CTGAN-generated synthetic data combined with a Random Forest classifier improved prediction accuracy to 98.93%.	Synthetic data augmentation improves lung cancer detection accuracy and manages class imbalance well.
Almahasneh et al. (2024)	AttentNet: Fully Convolutional 3D Attention for Lung Nodule Detection	Proposed AttentNet, improving lung nodule detection accuracy with 3D convolutional attention.	3D attention mechanisms enhance lung nodule detection in medical imaging.
Al-Jamimi et al. (2025)	Integrating Advanced Techniques: RFE-SVM Feature Engineering and Nelder–Mead Optimized XGBoost for Accurate Lung Cancer Prediction	Recursive Feature Elimination (RFE) with SVM improved feature selection, and Nelder–Mead optimized XGBoost achieved 100% accuracy.	Sophisticated feature engineering and hyperparameter tuning improve lung cancer classification.
Rahmanian and Mansoori (2024)	MoVAE: Multi-Omics Variational Auto-Encoder for Cancer Subtype Detection	MoVAE extracted and integrated multi-omics features for subtype classification, achieving superior accuracy.	Variational auto-encoders better classify cancer subtype using multi-omics data.
Chen et al. (2024)	Development of Lung Cancer Risk Prediction ML Models for an Equitable Learning Health System	XGBoost model with 29 features had 82% accuracy for risk-based lung cancer screening.	ML-based risk models improve access and accuracy for early lung cancer screening.
Liang (2025)	Using Synthetic Gaussian Noise to Explore Stochastic Resonance in Cancer Subtype Classification	Weak feature detection was enhanced by stochastic resonance to improve cancer subtype classification accuracy.	AI-based cancer diagnosis can be made better using controlled noise application.
Yang et al. (2022)	Machine Learning Application in Personalized Lung Cancer Recurrence and Survivability Prediction	Decision trees, neural networks, and SVMs were applied to predict recurrence and survival and identified genomic markers of importance.	ML can facilitate better personalized treatment planning and prognosis for lung cancer patients.
Yang et al. (2025)	Utilizing SMOTE-TomekLink and Machine Learning for Predictive Modeling	Applied SMOTE-Tomek Link with machine learning to improve prediction accuracy in elderly medical care demand.	Hybrid oversampling and undersampling techniques enhance ML-based predictive modeling for healthcare applications.
Dwivedi et al. (2023)	An Explainable AI-Driven Biomarker Discovery Framework for Non-Small Cell Lung Cancer Classification	Identified 52 key biomarkers using deep learning-based AI with 95.74% classification accuracy.	Explainable AI increases biomarker identification for targeted treatment of lung cancer.
Shadman et al. (2025)	A machine learning-based investigation of integrin expression patterns in cancer and metastasis	Machine learning algorithms were used for integrin expression patterns to investigate cancer development and metastasis.	Integrin expression is pivotal in cancer metastasis, and ML can contribute to pattern recognition for diagnosis.
Zhu et al. (2023)	Progressively Helical Multi-Omics Data Fusion GCN and Its Application in Lung Adenocarcinoma	Proposed a new GCN-based model to integrate multi-omics data for lung adenocarcinoma progression prediction.	Multi-omics integration improves accuracy of cancer classification and enhances patient stratification.
Jopek et al. (2024)	Deep Learning-Based Multiclass Approach to Cancer Classification on Liquid Biopsy Data	Used deep learning models to classify various types of cancer from liquid biopsy data with high accuracy.	Liquid biopsy-based classification via deep learning is an upcoming non-invasive cancer detection strategy.
McDowell et al. (2022)	Machine-learning algorithms for asthma, COPD, and lung cancer risk assessment using circulating microbial extracellular vesicle data	Formulated ML-based predictive models for lung diseases with high AUC scores (0.93–0.99).	Microbial extracellular vesicles can be used as biomarkers for lung disease risk prediction.
Wani et al. (2024)	DeepXplainer: An interpretable deep learning-based approach for lung cancer detection using explainable artificial intelligence	Proposed a hybrid CNN-XGBoost model for lung cancer detection with 97.43% accuracy.	Explainable AI improves lung cancer prediction confidence and facilitates clinical decisions.
Datta (2025)	Comparative investigation of lung adenocarcinoma and squamous cell carcinoma transcriptome to reveal potential candidate biomarkers	Identified key gene expressions differentiating LUAD and LUSC using XAI-based SHAP framework.	AI-powered biomarker discovery can enhance precision medicine for NSCLC subtypes.
Zhao et al. (2022)	GMILT: A Novel Transformer Network That Can Noninvasively Predict EGFR Mutation Status	Developed a transformer-based network to predict EGFR mutation status from CT scans, achieving AUC 0.772.	Transformer models can predict non-invasive mutations, supporting personalized therapy.
Mahum and Al-Salman (2023)	Lung-RetinaNet: Lung Cancer Detection Using a RetinaNet With Multi-Scale Feature Fusion and Context Module	Proposed a RetinaNet-based model that achieved 99.8% accuracy in lung cancer detection.	Multi-scale feature fusion enhances the early detection of lung cancer tumors.
Fadel et al. (2022)	A Fast Accurate Deep Learning Framework for Prediction of All Cancer Types	Developed an optimized LSTM model for predicting all cancer types with 100% accuracy.	Effective deep learning architectures can transform generalized cancer diagnosis.

### 2.1 Contributions of the research

This work presents a holistic method for lung cancer risk prediction through the combination of data augmentation methods with machine learning algorithms. Class imbalance is addressed and predictive accuracy is improved by rigorous experimentation across different preprocessing and classification approaches. The contributions of this work are as follows:

- Fine-tuned Augmentation Combinations: Unlike previous studies that apply a single resampling method, we experiment with multiple augmentation strategies to systematically evaluate their impact when paired with different classifiers. This directly addresses the lack of comparative analysis in prior research.
- Model-Specific Performance Evaluation: We demonstrate how each augmentation technique interacts with specific models, revealing that no single combination universally outperforms the rest. This adds critical nuance to the model selection process, extending beyond the one-size-fits-all approaches common in existing work.
- Explainability Focus with LIME Interpretability: While many studies prioritise accuracy, few incorporate model explainability. We use LIME to interpret predictions across all combinations, making our models more transparent and clinically trustworthy.
- Comprehensive Evaluation Framework: In addition to standard metrics like accuracy and F1-score, we incorporate AUC-ROC curves, confusion matrices, and LIME-based insights to give a holistic view of each method’s strengths and weaknesses. This multi-layered evaluation is lacking in prior literature and bridges the gap between performance and interpretability.

These nuances strengthen the research by making it not just a performance comparison but a systematic and interpretable framework for lung cancer risk prediction.

## 3 Proposed methodology

**Figure 1:**
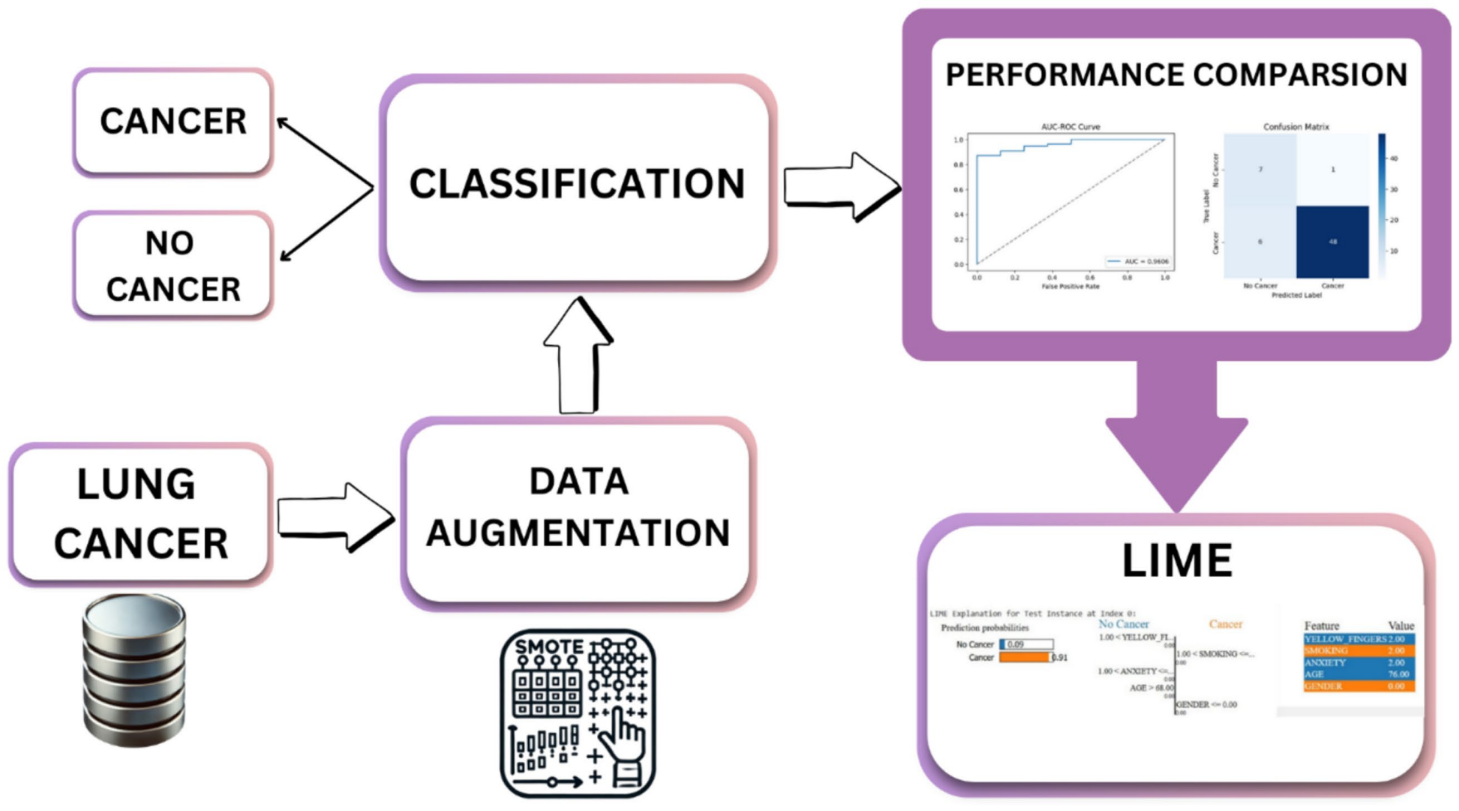
Proposed architecture of the methodology.

**Figure 2:**
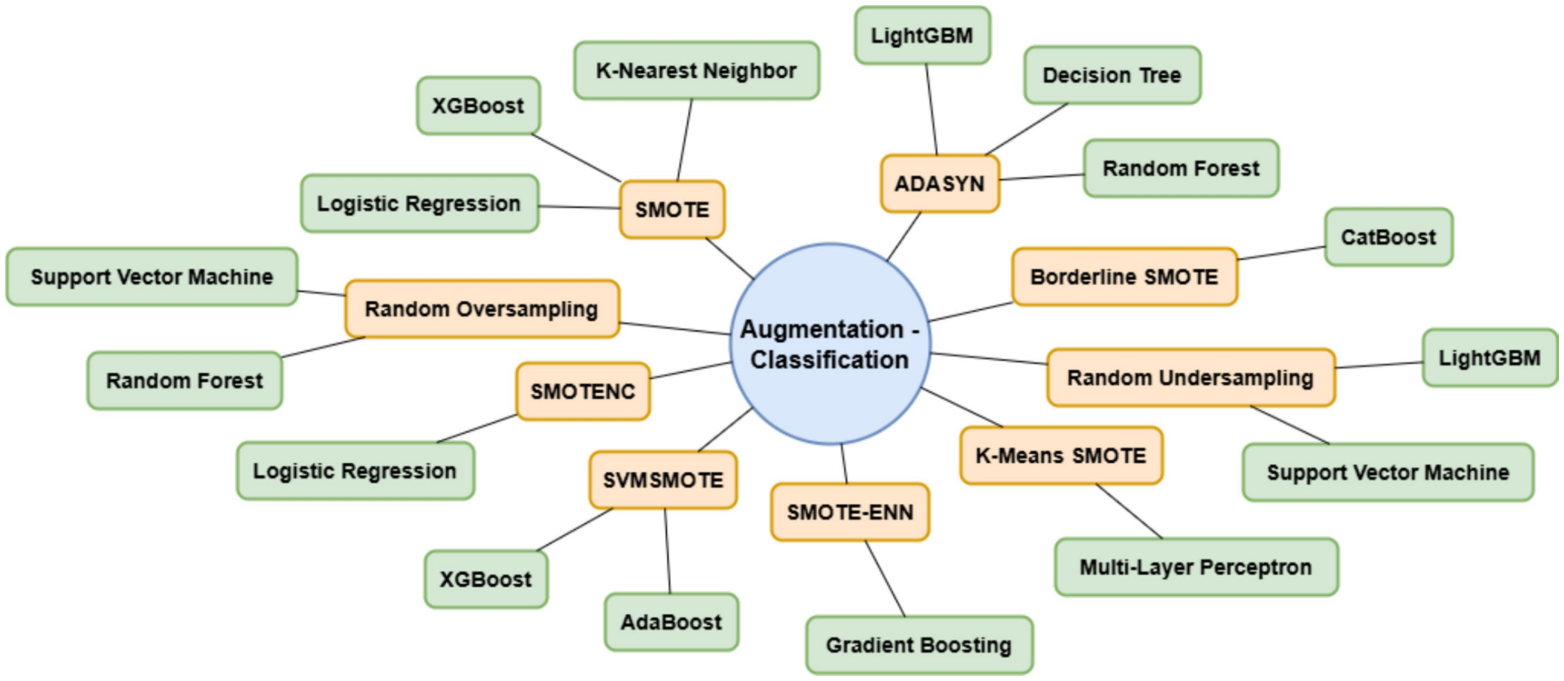
Combination of augmentation-classification methods used for lung cancer risk prediction.

The proposed methodology enhanced lung cancer risk prediction by integrating data augmentation, classification, and explainability. The process starts with a lung cancer dataset, in which synthetic samples are generated using various techniques to balance the class distribution. Augmented data are then fed into the classification models to improve prediction accuracy. A performance evaluation was conducted using various performance metrics, AUC-ROC curves, and confusion matrices. Finally, LIME was applied for interpretability and provided insights into feature importance. This architecture ensures robust predictions, mitigates bias from imbalanced data, and enhances model transparency for medical decision-making, as outlined in Figure 1. Figure 2 shows the combination of augmentation-classification methods used for lung cancer risk prediction.

### 3.1 Dataset overview

**Figure 3:**
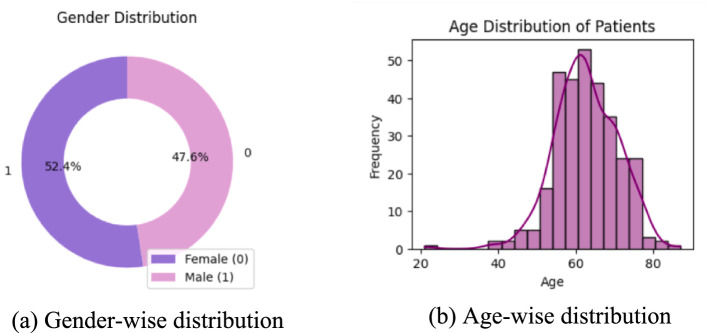
Age and gender distribution of the patients. **(a)** Donut chart showing gender distribution, with 52.4% female and 47.6% male. **(b)** Histogram displaying age distribution of patients.

**Figure 4:**
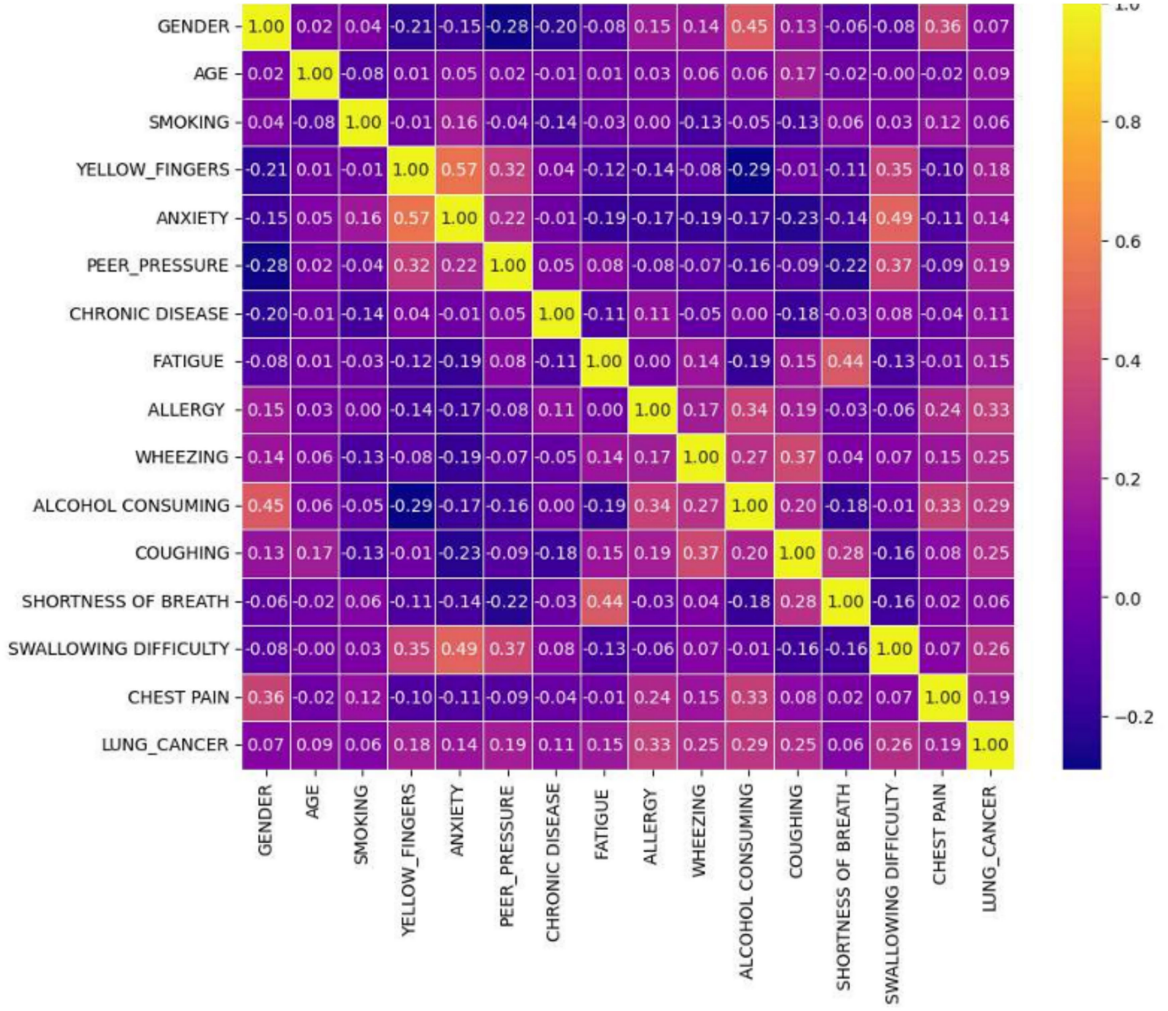
Feature correlation heatmap showing the relationships between different features.

**Figure 5:**
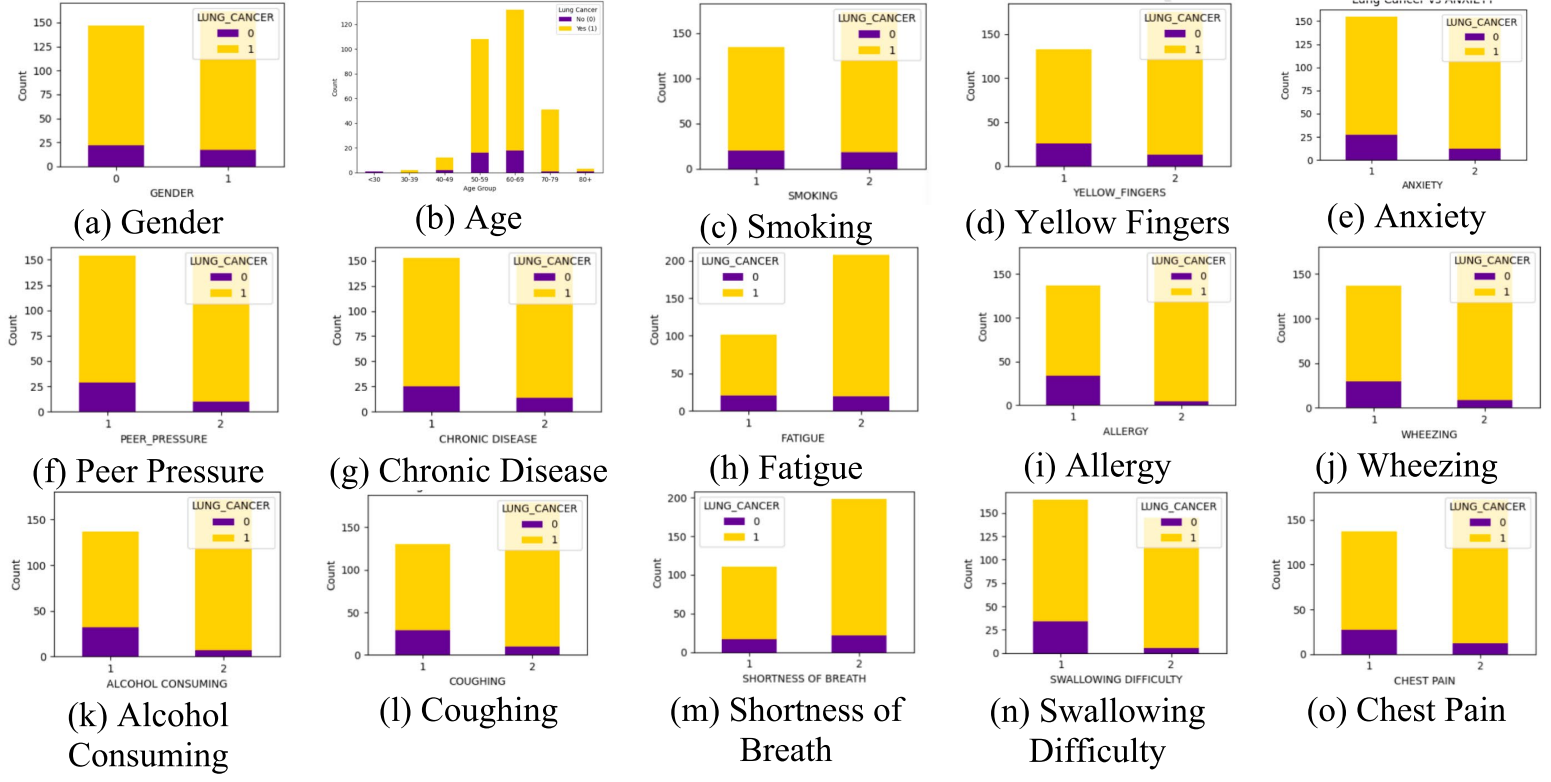
Stacked bar chart of each feature. **(a)** Gender, **(b)** Age, **(c)** Smoking, (**(d)** Yellow Fingers, **(e)** Anxiety, **(f)** Peer Pressure, **(g)** Chronic Disease, **(h)** Fatigue, **(i)** Allergy, **(j)** Wheezing, **(k)** Alcohol Consuming, **(l)** Coughing, **(m)** Shortness of Breath, **(n)** Swallowing Difficulty, **(o)** Chest Pain, with the target variable lung cancer.

Lung cancer is a significant health issue worldwide and is often linked to risk factors, such as smoking, age, and various respiratory conditions. The dataset taken from Kaggle for analysis contained 309 records and 16 attributes, providing information on different symptoms, habits, and patient demographics. The dataset included both categorical and numerical variables. The target variable, LUNG_CANCER, was labeled as YES/NO. Other variables included demographic factors such as GENDER, AGE, and lifestyle-related factors such as SMOKING, ALCOHOL CONSUMING, YELLOW_FINGERS, and PEER_PRESSURE; and health conditions such as COUGHING, SHORTNESS OF BREATH, FATIGUE, SWALLOWING DIFFICULTY, CHEST PAIN, ANXIETY, CHRONIC DISEASE, WHEEZING, and ALLERGY. The dataset is imbalanced, with more instances of lung cancer cases than non-cancer cases, as shown in Table 2. A closer examination of the gender distribution revealed that the dataset contained more male patients than female patients. Since smoking habits and lung cancer rates differ between genders, this imbalance might impact the predictive power of certain features. Additionally, the age distribution plot shows that most individuals in the dataset were middle-aged or older, which aligns with real-world data, as lung cancer is more prevalent in older populations. Figure 3 show the gender- and age-wise distributions of patients in the dataset. To identify the relationships between the features, a correlation heatmap was plotted, as depicted in Figure 4. The stacked bar charts in Figure 5 visualize the relationship between lung cancer and various features, including demographics, habits, and symptoms. They highlight the distribution of lung cancer cases across different feature categories, helping to identify strong influencing factors.

**Table 2:** Class distribution in patients with lung cancer in percentage.

Class	Yes [1]	No [0]
Percentage	87.45	12.55

### 3.2 Data preprocessing

In machine learning, data preprocessing is an essential phase that converts raw data into a format suitable for model training and evaluation. This research’s preprocessing encompassed data cleaning, encoding, addressing class imbalance, feature scaling, and dataset division. These steps collectively enhance model performance and to promote fair learning.

The dataset was examined for inconsistencies, null values, and structural issues. Although no missing values were identified, relevant transformations were used on categorical variables to allow model training. A central transformation was encoding categorical variables. Two columns in the dataset were categorical: GENDER and LUNG_CANCER. Since machine learning algorithms normally need numerical inputs, these variables were transformed into numerical representations. In particular, GENDER was encoded as 1 for Male and 0 for Female, whereas LUNG_CANCER was encoded as 1 for “YES” (cancer) and 0 for “NO” (no cancer). This encoding enables the model to handle these features precisely without being skewed by non-numeric values. After encoding, the dataset was split into features and target variables. Independent variables consisted of all the features except the target column, and the dependent variable is the LUNG_CANCER class label. This division allows the model to learn patterns in the feature set that help in the prediction of lung cancer risk. The dataset was divided with the common 80:20 train-test set ratio. This partitioning ensures sufficient data for pattern learning while reserving an adequate portion for assessing generalization performance.

One of the most significant issues in medical datasets is class imbalance. In this study, lung cancer-negative samples were much smaller than positive samples, and this had a tendency to bias the model toward the majority class (cancer). To address this issue, data augmentation techniques were employed to balance the data. For tabular data, this is synthetic data generation techniques that create more samples for the minority class, resulting in a better balanced dataset. The effectiveness of these augmentation techniques was validated by comparing the class distributions before and after the augmentation. Balancing the data improves the model’s ability to identify lung cancer-negative samples rather than overfitting on the majority class. After balancing the data, feature scaling was performed. Many machine learning methods, especially distance-based calculation-based algorithms, are improved by standardized input features. Standardization ensures numerical features have a mean of 0 and a standard deviation of 1, preventing features with larger numerical ranges from overpowering others. Scaling the data enhances model learning efficiency and avoids sensitivity to differences in numerical scales.

By these preprocessing steps, the data is converted to a form that is best suited for machine learning. These steps enable the model to generalize more to unseen data, enhance the accuracy of classification, and minimize bias, thus making the lung cancer risk prediction more accurate.

### 3.3 Augmentation and classification

Imbalanced datasets have a tendency to produce biased models that favor the majority class and thus generalize poorly for minority class samples. In a bid to solve this, numerous data augmentation techniques have been employed to counteract class imbalance in lung cancer risk prediction. By systematically augmenting techniques with classification models, the objective of this research is to improve the predictive accuracy without compromising a fair representation of both classes. This method gives an overall difference of the impact of various resampling methods in lung cancer risk estimation. Table 3 presents the combinations of different augmentation methods and classification systems used in this research and their justification. Equations 1–16 represent the mathematical formulae employed in each method.

**Table 3:** Hybrid of different augmentation technique and classification model.

S No.	Augmentation technique	Classification model	Mathematical equation	Justification
1	SMOTE	Logistic Regression	Synthetic sample generation: $X_{\mathrm{new}}=X_{\min}+\lambda \cdot \left(X_{\mathrm{neighbor}}-X_{\min}\right)$ (1) Equation 1 represents the synthetic sample generation process in SMOTE. In this approach, a new synthetic minority instance $X_{\mathrm{new}}$ is generated by interpolating between an actual minority sample $X_{\min}$ and one of its k-nearest neighbors $X_{\mathrm{neighbor}}$, where λ∈[0,1] is a random scalar. This interpolation creates new data points that lie along the line segments joining each minority sample and its neighbors in feature space. This helps fill the sparse regions of the minority class, addressing class imbalance and allowing machine learning models to learn more generalizable decision boundaries.	SMOTE balances the dataset by generating synthetic minority samples, which is crucial for Logistic Regression. Without balancing, Logistic Regression, a linear model, would be heavily biased toward the majority class in an imbalanced medical dataset, leading to poor performance on identifying the rare, but critical, positive cases. SMOTE ensures unbiased predictions by providing the model with a more representative view of both classes.
2	SMOTE	K-Nearest Neighbor	KNN Distance Calculation: $D\left(W_{knn},W_{train}\right)=\sqrt{\sum_{k=1}^{p}{\left(w_{k}-w_{k}^{\prime }\right)}^{2}}$ (2) Equation 2 represents the Euclidean distance used in the KNN classifier. For a given input vector $W_{knn}$, the distance is computed from all training instances $W_{train}$ using their feature differences across pp dimensions. The model then selects the k closest training instances based on this distance. In this study, KNN is applied after SMOTE-based augmentation to ensure that the nearest neighbors include a balanced representation of both classes. This enhances minority class recognition during classification by avoiding the majority class dominance that typically skews KNN performance in imbalanced datasets.	KNN is a distance-based algorithm. In imbalanced datasets, the minority class instances are sparse, and their neighbors are often from the majority class. This can cause KNN to misclassify minority instances. SMOTE directly addresses this by creating synthetic minority samples, thus increasing the density of the minority class and ensuring that KNN has enough minority samples nearby to make accurate classifications, preventing it from favoring the majority class.
3	SMOTE	XGBoost	XGBoost Additive Model Update: ${\hat{y}}_{i}^{\left(t\right)}={\hat{y}}_{i}^{\left(t-1\right)}+f_{t}\left(x_{i}\right)$ (3) Equation 3 describes the boosting process in XGBoost, where predictions are updated iteratively. At each boosting round t, a new decision tree $f_{t}$ is trained to predict the residual errors from the previous round t−1. The new prediction ${\hat{y}}_{i}^{\left(t\right)}$ is obtained by adding the new tree’s output to the prior prediction. In this study, XGBoost is paired with SMOTE to reduce class imbalance, enabling each successive tree to focus more effectively on difficult minority class examples. This additive strategy improves model generalization and performance, particularly in noisy or skewed clinical datasets.	XGBoost is an ensemble tree-based algorithm known for its strong performance. While robust, it can still be affected by extreme class imbalance. SMOTE helps by providing a balanced dataset, allowing XGBoost to focus its learning on differentiating between the true patterns of both classes rather than being overwhelmed by the majority class. The combination helps XGBoost generalize better and reduce misclassifications of the minority class.
4	ADASYN	Decision Tree	ADASYN Sample Generation: $G_{i}=r_{i}\times G\;$ (4) Equation 4 represents the adaptive sample generation strategy used in ADASYN. Unlike SMOTE, which generates a fixed number of synthetic samples for all minority instances, ADASYN uses a dynamic approach where $G_{i}$ depends on the local difficulty $r_{i}$ of learning a specific minority instance. The higher the local class imbalance near a sample, the more synthetic samples it receives. This adaptiveness allows ADASYN to focus on the harder-to-classify regions of the decision boundary. In this study, ADASYN was combined with Decision Tree and Random Forest models to observe its effect on ensemble classifiers under high-variance conditions.	ADASYN generates synthetic samples for minority class instances that are harder to learn. Decision Trees can create complex, non-linear decision boundaries. By generating samples in complex regions, ADASYN helps the Decision Tree build more robust and accurate splits, particularly for the minority class, improving its ability to classify those instances
5	ADASYN	Random Forest	Random Forest Prediction: $\hat{z}=\frac{1}{N}\sum_{j=1}^{N}f_{j}\left(W_{rf}\right)$ (5) Equation 5 illustrates how Random Forest aggregates predictions from multiple decision trees. Each individual tree $f_{j}$ makes a prediction on the input $W_{rf}$, and the final output $\hat{z}$ is computed by averaging the outputs across all N trees. In classification tasks, this often corresponds to majority voting, whereas in probabilistic outputs, it may represent the mean predicted probability. The averaging mechanism in Random Forest makes it robust to noise and overfitting, especially when augmented data introduces synthetic variability.	Random Forest, an ensemble of Decision Trees, can also struggle with imbalanced data, as each tree might be biased toward the majority class. ADASYN ensures that the Random Forest trains on a balanced dataset. This prevents individual trees from overfitting to the majority class and helps them learn more effectively from the minority class, leading to improved overall generalization.
6	ADASYN	LightGBM	Gradient-Based Learning: $L=\sum_{i=1}^{N}l\left(y_{i},{\hat{y}}_{i}\right)+\lambda \sum_{j=1}^{M}w_{j}^{2}$ (6) Equation 6 represents the regularized loss function used in gradient-based learners like LightGBM. The first term $\sum l\left(y_{i},{\hat{y}}_{i}\right)$ computes the loss between actual and predicted labels using a suitable function, while the second term $\lambda \sum w_{j}^{2}$ penalizes model complexity by discouraging overly large parameter weights through L2 regularization. This combination enables the model to fit the data effectively while avoiding overfitting. Regularization ensures that even when synthetic samples are added, the model maintains generalizability and robustness to noise.	LightGBM is a gradient-boosting framework known for its speed and efficiency. In imbalanced datasets, the gradients for the minority class might be small or overshadowed by the majority class. ADASYN’s focus on generating samples for hard-to-learn minority instances helps LightGBM assign more significant weight to these crucial samples, enabling it to recognize minority class patterns efficiently during its gradient-based learning process.
7	SVMSMOTE	XGBoost	Support Vector-Based Sample Generation: $X_{\mathrm{new}}=X_{\mathrm{sv}}+\lambda \cdot \left(X_{\mathrm{neighbor}}-X_{\mathrm{sv}}\right)$ (7) Equation 7 describes the sample generation process in SVMSMOTE, a technique that focuses on more informative regions of the decision boundary. Instead of randomly choosing any minority instance, SVMSMOTE identifies support vectors of the minority class using a support vector machine and generates synthetic samples by interpolating between those support vectors and their nearby minority neighbors. The generated points $X_{\mathrm{new}}$ are more likely to lie close to complex boundary regions, enhancing the model’s ability to distinguish between classes.	SVMSMOTE is a variant of SMOTE that focuses on generating synthetic samples in regions near the support vectors of a Support Vector Machine, which are often critical decision boundaries. By placing synthetic samples strategically near these boundaries, SVMSMOTE provides XGBoost with more informative data points. This helps XGBoost refine its decision boundaries, especially in the nuanced areas where the minority and majority classes are hard to distinguish.
8	SVMSMOTE	AdaBoost	Weight Update in AdaBoost: $w_{i}^{\left(t+1\right)}=w_{i}^{\left(t\right)}e^{-\alpha_{t}y_{i}f_{t}\left(X_{i}\right)}$ (8) Equation 8 shows how instance weights are updated in the AdaBoost algorithm. After each round t, misclassified instances receive increased weights, while correctly classified ones are down-weighted, based on the classifier’s performance. The parameter $\alpha_{t}$ reflects the influence of the current weak learner, and the exponential term ensures that harder-to-classify instances become more influential in the next iteration. In this study, AdaBoost is combined with SVMSMOTE to focus on generating synthetic samples near the decision boundary while adaptively learning from difficult examples. This synergy helps the ensemble progressively reduce classification error, especially for underrepresented lung cancer-negative cases in an imbalanced dataset.	AdaBoost works by iteratively focusing on misclassified samples, weighting them more in subsequent iterations. When dealing with imbalanced data, AdaBoost might struggle to give enough attention to the minority class. SVMSMOTE, by generating synthetic samples near the decision boundaries, essentially provides AdaBoost with more misclassified minority instances. This allows AdaBoost to iteratively reduce errors in minority class predictions by focusing its boosting efforts more effectively on the minority class.
9	Borderline SMOTE	CatBoost	Borderline SMOTE Sample Generation: $X_{border}=X_{b}+\delta \left(X_{near}-X_{b}\right)$ (9) Equation 9 illustrates how Borderline SMOTE generates synthetic samples near the decision boundary, where misclassification is most likely to occur. It selects minority class instances that are surrounded by many majority class neighbors, these are considered at risk. New samples are then created by interpolating between these borderline instances $X_{b}$ and their nearby minority neighbors $X_{\mathrm{near}}$. This helps sharpen the class boundary by reinforcing decision-making in complex regions. In this study, Borderline SMOTE is paired with CatBoost, a gradient boosting classifier known for handling categorical variables, to evaluate how boundary-focused augmentation affects predictive precision in imbalanced lung cancer datasets.	Borderline SMOTE focuses on generating synthetic samples for minority instances that are borderline – meaning they are close to the decision boundary and thus more prone to misclassification. CatBoost is another powerful gradient-boosting algorithm. By providing CatBoost with these crucial borderline samples, Borderline SMOTE helps the model learn more precisely around the critical classification boundaries, thereby improving CatBoost’s classification by ensuring it does not overlook these ambiguous minority instances.
10	SMOTENC	Logistic Regression	SMOTENC Sample Generation: $X_{\mathrm{new}}=X_{\mathrm{cat}}+\lambda \cdot \left(X_{\mathrm{neighbor}}-X_{\mathrm{cat}}\right)$ (10) Equation 10 shows how SMOTENC generates synthetic samples when both numerical and categorical variables are present. For numerical features, interpolation is performed in the same way as SMOTE. However, for categorical features, SMOTENC selects values using a majority vote, ensuring logical consistency in the synthetic data. This is particularly important in medical datasets where features like gender or smoking status are categorical and should not be interpolated. In this study, SMOTENC is used with logistic regression to evaluate how well classical interpretable models perform when both balanced and semantically valid synthetic data are introduced.	SMOTENC is specifically designed for datasets with a mix of categorical and numerical features. The lung cancer dataset contains both. Logistic Regression needs its input features to be handled appropriately, especially categorical ones. SMOTENC ensures that when synthetic samples are generated, both the numerical and categorical features are augmented in a way that maintains their relationships and balance. This leads to fair logistic regression predictions by accurately representing both types of features and preventing bias.
11	K-Means SMOTE	Multi Layer Perceptron	K-Means SMOTE Sample Generation: $X_{cluster}=X_{c}+\alpha \left(X_{near}-X_{c}\right)$ (11) Equation 11 shows how K-Means SMOTE enhances the standard SMOTE process by incorporating clustering before sample generation. Minority class samples are grouped into clusters using K-Means, and synthetic points are generated by interpolating between the cluster centroid $X_{c}$ and neighboring minority instances within the same cluster. This approach ensures that new samples are created in dense, meaningful regions of the feature space, reducing the risk of generating outliers or noisy data.	K-Means SMOTE first clusters the minority class instances using K-Means and then applies SMOTE within these clusters, generating synthetic samples in more meaningful regions. By providing the Multi-Layer Perceptron with well-clustered and augmented minority samples, K-Means SMOTE enhances Multi-Layer Perceptron’s performance. This helps the Multi-Layer Perceptron to better distinguish between the classes, particularly in areas where minority samples might otherwise be scarce or outliers.
12	SMOTE-ENN	Gradient Boosting	SMOTE-ENN Sample Generation: $X_{clean}=X_{resampled}-X_{noisy}\;$ (12) Equation 12 captures the two-step process in SMOTE-ENN: oversampling followed by data cleaning. First, SMOTE is applied to generate synthetic minority class samples, resulting in $X_{\mathrm{resampled}}$. Then, ENN filters out both synthetic and original samples that are misclassified by their neighbors, these are labeled as $X_{\mathrm{noisy}}$. Subtracting these gives the final cleaned dataset $X_{\mathrm{clean}}$, which is used to train the model. This hybrid approach improves both class balance and data quality. In this study, SMOTE-ENN is paired with Gradient Boosting to assess whether removing noisy data post-augmentation enhances the generalization ability of ensemble learners in lung cancer risk prediction.	SMOTE-ENN combines SMOTE with Edited Nearest Neighbors. After SMOTE generates synthetic samples, ENN removes instances that are misclassified by their neighbors, essentially cleaning up noisy or ambiguous data points. Gradient Boosting is a powerful ensemble method that builds models sequentially, correcting errors from previous models. By using SMOTE-ENN, the Gradient Boosting model is trained on a cleaner, balanced dataset, where noisy data that could hinder learning has been removed while balancing classes, thereby improving Gradient Boosting accuracy.
13	Random Oversampling	Random Forest	Random Oversampling Sample Generation: $X_{over}=X_{rand}\cup X_{rand\_dup}$ (13) Equation 13 describes how Random Oversampling balances the dataset by randomly duplicating minority class instances. A subset of the existing minority samples $X_{\mathrm{rand}}$ is selected and replicated to form $X_{rand\_\mathrm{dup}}$, and the two are combined to create an oversampled dataset $X_{\mathrm{over}}$. Although this method does not introduce new variability like SMOTE, it ensures that the classifier does not become biased toward the majority class.	Random Oversampling simply duplicates random instances of the minority class. While simpler than SMOTE, it directly increases the number of minority samples. Random Forest is an ensemble method that can benefit from a larger and more balanced training set. By increasing minority representation through Random Oversampling, the individual trees within the Random Forest are more likely to encounter minority samples during training, allowing the Random Forest to generalize better to both classes and reducing bias toward the majority.
14	Random Oversampling	Support Vector Machine	SVM Decision Function: $f\left(X\right)=w^{T}X+b$ (14) Equation 14 defines the linear decision function used in Support Vector Machines. The function f(X) computes a score by projecting the input vector X onto the learned weight vector ww and adding a bias term b. The sign of f(X) determines the class label. This formulation allows the SVM to find an optimal hyperplane that maximizes the margin between classes.	Support Vector Machines aim to find an optimal hyperplane that separates classes with the maximum margin. In imbalanced datasets, the SVM’s hyperplane can be skewed toward the majority class, as there are fewer minority samples to define its boundary. Random Oversampling increases the presence of minority samples, providing SVM with more data points from the minority class to establish a more robust and balanced decision boundary for both classes.
15	Random Undersampling	Support Vector Machine	Random Undersampling Sample Generation: $X_{under}=X_{rand}-X_{rand\_del}$ (15) Equation 15 illustrates how Random Undersampling creates a balanced dataset by removing excess majority class instances. From the original majority dataset $X_{\mathrm{rand}}$, a subset $X_{rand\_\mathrm{del}}$ is randomly selected and discarded, leaving $X_{\mathrm{under}}$ for model training. While simple, this method reduces training time and bias toward the majority class but risks discarding potentially informative data.	Random Undersampling involves randomly removing instances from the majority class to balance the dataset. For SVMs, which are sensitive to the distribution of data points, reducing the overwhelming presence of the majority class can be beneficial. By balancing the dataset this way, Random Undersampling prevents SVM from being biased toward the majority class and enables it to learn a more effective decision boundary that considers both classes fairly.
16	Random Undersampling	LightGBM	LightGBM Prediction: $\hat{z}=\sum_{s=1}^{S}\beta_{s}g_{s}\left(W_{lgb}\right)$ (16) Equation 16 describes the prediction mechanism in LightGBM. The final output $\hat{z}$ is obtained by summing the predictions from multiple weak learners $g_{s}$, each weighted by a learning rate $\beta_{s}$. Each tree $g_{s}$ is trained sequentially to correct the residuals of the previous trees, allowing the model to iteratively refine its predictions.	Similar to SVM, LightGBM can also benefit from a more balanced dataset, and for very large datasets, undersampling can also improve training efficiency. Random Undersampling reduces the size of the majority class, which can speed up LightGBM training by reducing the overall data volume, while still preserving essential class patterns needed for effective learning. This approach aims for both improved balance and computational efficiency.

The augmentation techniques were selected based on their unique strengths in handling class imbalance in structured tabular datasets. SMOTE was used as a baseline due to its widespread applicability, while Borderline SMOTE was included to generate samples near decision boundaries, especially useful in medical datasets where minority instances are critical. SMOTENC was chosen to handle categorical features, which are common in clinical records. K-Means SMOTE enhances cluster-based generation of synthetic samples, and SMOTE-ENN combines oversampling with cleaning of noisy data for higher accuracy. These techniques offer a diverse range of behaviors in minority class modeling, allowing a thorough performance comparison.

### 3.4 Cross validation and HYPERPARAMETER optimisation

To ensure statistical reliability, all experiments were conducted using 5-fold stratified cross-validation, preserving the original class imbalance within each fold. The reported performance metrics (accuracy, precision, recall, F1-score, and AUC-ROC) represent average values across all folds. Additionally, we report 95% confidence intervals for each metric to quantify the variability across folds. For reproducibility, all models were trained using the same random seed, and train-test splits were stratified with an 80:20 ratio during initial hold-out evaluation. All augmentation techniques were applied only to the training data to avoid test set contamination. A 5-fold stratified cross-validation strategy was employed to balance computational efficiency with robust performance estimation, given the dataset’s limited size of 309 samples. Stratified sampling was used during 5-fold cross-validation to preserve the original class distribution within each fold. To prevent data leakage, data augmentation was applied exclusively to the training portion of each fold, with the validation set remaining untouched for unbiased evaluation. For reproducibility, all experiments used a fixed random seed of 42 for data splitting, augmentation, and model training, ensuring consistent results across runs.

For each classifier, we conducted hyperparameter optimization using a grid search within a 5-fold cross-validation framework on the training data. This approach systematically explored combinations of key hyperparameters, such as n estimators, max depth, and learning rate for tree-based models, and kernel and gamma for SVM, to identify configurations that balanced performance and generalization. Given the limited dataset size, we constrained the grid search space to avoid overfitting and model instability, focusing on achieving robust cross-validated performance rather than exhaustive optimization.

The Table 4 outlines the hyperparameter search space and selection criteria used for optimizing each classifier within the 5-fold cross-validation framework. For each classifier, key hyperparameters are listed along with their tested ranges, tailored to balance model complexity and performance on the imbalanced lung cancer dataset. For instance, Logistic Regression explores a range of regularization strengths to prevent overfitting, while tree-based models like XGBoost, Random Forest, and LightGBM test various tree counts, depths, and learning rates to optimize ensemble learning. The selection criterion, primarily the highest accuracy, F1-score or AUC-ROC, reflects the study’s focus on metrics suitable for imbalanced datasets, ensuring models prioritize balanced performance over mere accuracy. This table specifies the hyperparameter optimization process, providing transparency and reproducibility by detailing the configurations explored and the rationale for selecting the best-performing settings.

**Table 4:** Hyperparameter search space and selection criteria for the classifiers used.

Classifier	Hyperparameters	Search space	Selection criterion
Logistic Regression	C (inverse regularization strength)	[0.01, 0.1, 1, 10, 100]	Highest F1-score
K-Nearest Neighbor	n_neighbors, weights	[3, 5, 7, 9], [‘uniform’, ‘distance’]	Highest AUC-ROC
XGBoost	n_estimators, max_depth, learning_rate	[50, 100, 200], [3, 5, 7], [0.01, 0.1, 0.3]	Highest F1-score
Decision Tree	max_depth, min_samples_split	[3, 5, 7], [2, 5, 10]	Highest F1-score
Random Forest	n_estimators, max_depth, min_samples_split	[50, 100, 200], [3, 5, 7], [2, 5, 10]	Highest F1-score
LightGBM	n_estimators, max_depth, learning_rate	[50, 100, 200], [3, 5, 7], [0.01, 0.1, 0.3]	Highest F1-score
AdaBoost	n_estimators, learning_rate	[50, 100, 200], [0.01, 0.1, 1.0]	Highest F1-score
CatBoost	iterations, depth, learning_rate	[50, 100, 200], [3, 5, 7], [0.01, 0.1, 0.3]	Highest F1-score
Multi-Layer Perceptron	hidden_layer_sizes, learning_rate_init	[(50,50), (100,50), (100,100)], [0.001, 0.01, 0.1]	Highest AUC-ROC
Support Vector Machine	C, kernel, gamma	[0.1, 1, 10], [‘rbf’, ‘linear’], [‘scale’, ‘auto’, 0.1, 1]	Highest AUC-ROC
Gradient Boosting	n_estimators, max_depth, learning_rate	[50, 100, 200], [3, 5, 7], [0.01, 0.1, 0.3]	Highest F1-score

### 3.5 Evaluation metrics

Several evaluation metrics are employed to compare each augmentation-classification approach for lung cancer risk prediction effectively. Accuracy is the measure of the proportion of cases correctly classified. With the calculation of the proportion of actual lung cancer cases among predicted positives, precision eliminates false positives. Recall estimates the model’s capacity to identify true instances of lung cancer without omitting any false negatives. Precision measures and recall balance through the F1-score to maximize categorization. The AUC-ROC curve illustrates sensitivity and specificity trade-offs, and the AUC-ROC score estimates the model’s capacity to discriminate between cancer and non-cancer. To deliver an extensive performance analysis toward medical decision-making, the confusion matrix offers a comprehensive differentiation of true and false classification.

### 3.6 Explainable AI

It is significant to acknowledge model choices in clinical reproducibility and acceptability in lung cancer classification. In a balanced feature importance comparison, it uses index = 0 from the test set to interpret single predictions for all methods. This provides interpretability with varying augmentation and classification methods through guaranteeing transparency in models. LIME provides multiple outputs that are beneficial in explanation of the prediction:

- Prediction Probabilities - Displays the model’s confidence in different classes.
- Feature Contributions - Visual representation of how individual features influence the prediction.
- Feature Importance Table - Lists the top contributing features with their corresponding values.

## 4 Results and analysis

The results are presented in terms of several evaluation metrics for comparison across various methodologies. Class distribution after data augmentation is shown to emphasize the effect of resampling techniques. AUC-ROC curves are used for depicting model’s class separating ability in graphical form. Confusion matrices give a clear idea of classification accuracy through a representation of correct and incorrect predictions. LIME explanations are provided for individual predictions to mark feature importance and enhance the explainability of the model. An overall view of methodologies carried out is shown with the inclusion of these evaluations.

A well-balanced dataset avoids model bias, resulting in accurate and unbiased predictions. Table 5 shows how resampling methods avoid class imbalances from being handled prior to training.

**Table 5:** Class distribution after various augmentation techniques in percentage.

Augmentation technique	Classification model	Class	Distribution
		YES [1]	NO [0]
SMOTE	Logistic Regression	50.00	50.00
	K-Nearest Neighbor	50.00	50.00
	XGBoost	50.00	50.00
ADASYN	Decision Tree	49.42	50.58
	Random Forest	49.42	50.58
	LightGBM	50.35	49.65
SVMSMOTE	XGBoost	61.90	38.10
	AdaBoost	61.90	38.10
Borderline SMOTE	CatBoost	50.00	50.00
SMOTENC	Logistic Regression	50.00	50.00
K-Means SMOTE	Multi Layer Perceptron	49.77	50.23
SMOTE-ENN	Gradient Boosting	50.00	50.00
Random oversampling	Random Forest	50.00	50.00
	Support Vector Machine	50.00	50.00
Random undersampling	Support Vector Machine	66.66	33.34
	LightGBM	66.66	33.34

Key performance metrics quantify model effectiveness. They provide a numerical assessment of classification performance, ensuring a comprehensive comparison of different methods. Table 6 reports average performance metrics across 5 folds, along with their 95% confidence intervals.

**Table 6:** A comparison of performance metrics for different methods applied over lung cancer in percentage.

Augmentation	Classification	Accuracy	Precision	Recall	F1 Score	AUC-ROC Score
SMOTE	Logistic Regression	87.10	97.92	87.04	92.16	94.91
	K-Nearest Neighbor	87.10	97.92	87.04	92.16	90.51
	XGBoost	91.94	98.04	95.24	92.59	95.83
ADASYN	Decision Tree	88.71	96.08	90.74	93.33	82.87
	Random Forest	91.94	98.04	92.59	95.24	94.56
	LightGBM	88.71	96.08	90.74	93.33	96.30
SVMSMOTE	XGBoost	88.71	96.08	90.74	93.33	92.59
	AdaBoost	88.71	92.73	94.44	93.58	93.40
Borderline SMOTE	CatBoost	90.32	96.15	92.59	94.34	93.98
SMOTENC	Logistic Regression	88.71	94.34	92.59	93.46	95.37
K-Means SMOTE	Multi-Layer Perceptron	93.55	98.08	94.44	96.23	96.76
SMOTE-ENN	Gradient Boosting	88.71	100.00	87.04	93.07	94.91
Random oversampling	Random Forest	90.32	96.15	92.59	94.34	95.02
	Support Vector Machine	88.71	97.96	88.89	93.20	96.06
Random undersampling	Support Vector Machine	87.10	97.92	87.04	92.16	93.98
	LightGBM	88.71	100.00	87.04	93.07	93.98

Among all combinations, K-Means SMOTE paired with Multi-Layer Perceptron achieves the highest performance of 93.55% accuracy and 96.76% AUC-ROC. This combination is particularly effective for two reasons. First, K-Means SMOTE generates synthetic samples within minority clusters, preserving local density and reducing noise compared to traditional SMOTE. This ensures that the Multi-Layer Perceptron receives well-distributed training data. Second, Multi-Layer Perceptron’s non-linear architecture enables it to capture complex interactions between features, such as overlapping symptoms or comorbidities, which are common in lung cancer risk profiles. The synergy of structured sampling and high model capacity makes this pairing well-suited for the dataset’s imbalanced yet feature-rich nature.

While accuracy offers a broad view of model performance, it can be misleading in the context of imbalanced datasets. Therefore, we emphasize metrics such as precision, recall (sensitivity), F1 score, and AUC-ROC, which better capture the classifier’s ability to correctly identify the minority class and avoid false negatives. The highest recall values (94.44%) were observed for K-Means SMOTE + Multi-Layer Perceptron and SVMSMOTE + AdaBoost, indicating strong sensitivity, a critical factor in cancer risk prediction. Precision, which reflects the proportion of true positives among all predicted positives, reached 100% for SMOTE-ENN + Gradient Boosting and Random Undersampling + LightGBM, meaning these models were highly confident in their predictions, though potentially at the cost of missing some cases. The F1 score, a harmonic mean of precision and recall, was maximized by K-Means SMOTE + Multi-Layer Perceptron (96.23), highlighting it as the most balanced and robust combination. The AUC-ROC, which measures the model’s ability to distinguish between classes across thresholds, also peaked at 96.76 for this combination. These findings suggest that evaluating multiple metrics is essential for identifying models that are not only accurate but also clinically reliable in identifying high-risk patients.

**Figure 6:**
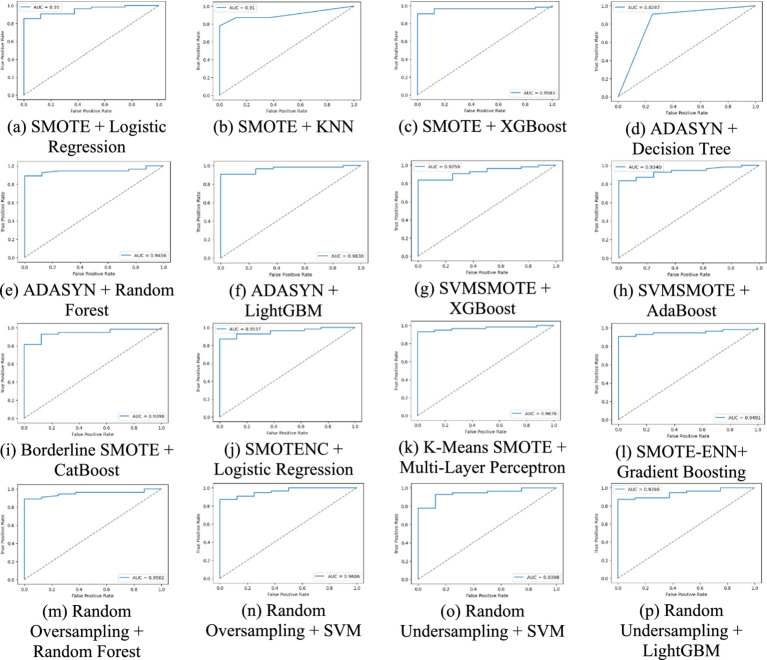
A comparative analysis of each method using AUC-ROC curve for lung cancer risk prediction. **(a)** SMOTE + Logistic Regression, **(b)** SMOTE + KNN, **(c)** SMOTE + XGBoost, **(d)** ADASYN + Decision Tree, **(e)** ADAYSN + Random Forest, **(f)** ADASYN + LightGBM, **(g)** SVMSMOTE + XGBoost, **(h)** SVMSMOTE + AdaBoost, **(i)** Borderline SMOTE + CatBoost, **(j)** SMOTENC + Logistic Regression, **(k)** K-Means SMOTE + Multi-Layer Perceptron, **(l)** SMOTE-ENN + Gradient Boosting, **(m)** Random Oversampling + Random Forest, **(n)** Random Oversampling + SVM, **(o)** Random Undersampling + SVM, **(p)** Random Undersampling + LightGBM.

Figure 6 graphs the AUC-ROC curves for all the methods, indicating the true positive vs. false positive trade-off. The larger the AUC score, the more the classes are well-separated, and therefore this is a valuable tool in classifier comparison.

**Figure 7:**
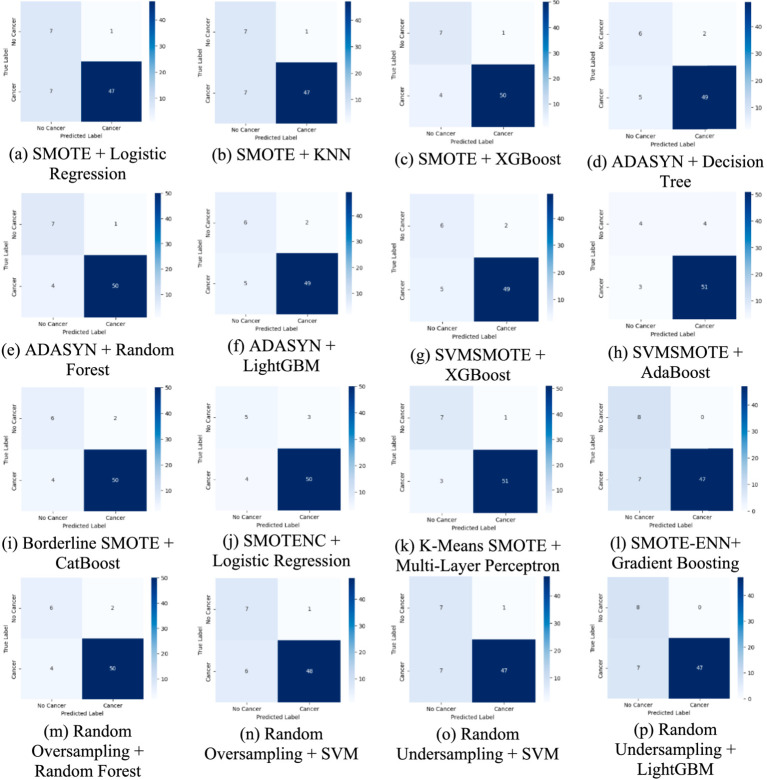
A comparative analysis of each method using confusion matrices for lung cancer risk prediction. **(a)** SMOTE + Logistic Regression, **(b)** SMOTE + KNN, **(c)** SMOTE + XGBoost, **(d)** ADASYN + Decision Tree, **(e)** ADAYSN + Random Forest, **(f)** ADASYN + LightGBM, **(g)** SVMSMOTE + XGBoost, **(h)** SVMSMOTE + AdaBoost, **(i)** Borderline SMOTE + CatBoost, **(j)** SMOTENC + Logistic Regression, **(k)** K-Means SMOTE + Multi-Layer Perceptron, **(l)** SMOTE-ENN + Gradient Boosting, **(m)** Random Oversampling + Random Forest, **(n)** Random Oversampling + SVM, **(o)** Random Undersampling + SVM, **(p)** Random Undersampling + LightGBM.

Figure 7 presents the confusion matrix, which provides an in-depth analysis of classification outcomes by detailing TP, FP, TN, and FN. It assists in identifying class-specific misclassifications, aiding in performance refinement.

The confusion matrices provide a detailed breakdown of model predictions. For weaker combinations, such as ADASYN + Decision Tree, we observe a higher number of false negatives, meaning the model fails to detect actual lung cancer cases, a critical issue in clinical settings. On the other hand, models like K-Means SMOTE + Multi-Layer Perceptron and SMOTE + XGBoost show improved true positive and true negative rates, indicating better generalization. Interestingly, Random Undersampling + SVM maintains low false positives but at the cost of higher false negatives, reflecting its conservative decision boundary due to reduced training size. These differences suggest that the choice of augmentation impacts not just accuracy but also error type, which is vital in medical diagnosis, where false negatives can delay treatment.

**Figure 8:**
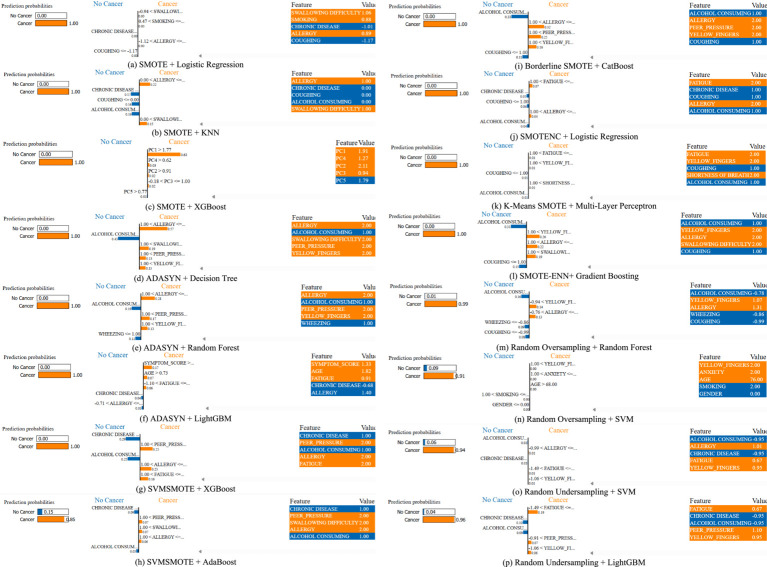
A comparative analysis for a specific instance of each method using LIME for lung cancer risk prediction. **(a)** SMOTE + Logistic Regression, **(b)** SMOTE + KNN, **(c)** SMOTE + XGBoost, **(d)** ADASYN + Decision Tree, **(e)** ADAYSN + Random Forest, **(f)** ADASYN + LightGBM, **(g)** SVMSMOTE + XGBoost, **(h)** SVMSMOTE + AdaBoost, **(i)** Borderline SMOTE + CatBoost, **(j)** SMOTENC + Logistic Regression, **(k)** K-Means SMOTE + Multi-Layer Perceptron, **(l)** SMOTE-ENN + Gradient Boosting, **(m)** Random Oversampling + Random Forest, **(n)** Random Oversampling + SVM, **(o)** Random Undersampling + SVM, **(p)** Random Undersampling + LightGBM.

Figure 8 provides LIME visualizations for a specific test instance. It demonstrates how each feature contributes positively or negatively to the classification outcome. These explanations illustrate how individual features influence predictions, enhancing model transparency and trustworthiness. LIME consistently highlighted clinically relevant features such as allergy, yellow fingers, and fatigue, aligning with established risk factors reported in clinical studies.

While LIME explanations provide general feature importance, we further analyzed index = 0 from the test set across different models to highlight patterns. For instance, in the K-Means SMOTE + Multi-Layer Perceptron case, the top contributing features included fatigue, yellow fingers and shortness of breath, all positively weighted toward lung cancer prediction. This aligns with known clinical risk factors. These LIME results indicate that augmentation strategies not only affect accuracy but also shape how models interpret risk, which is crucial for clinician trust and transparency. Such interpretability helps identify whether models are overfitting to shallow cues or capturing medically relevant risk patterns.

**Figure 9:**
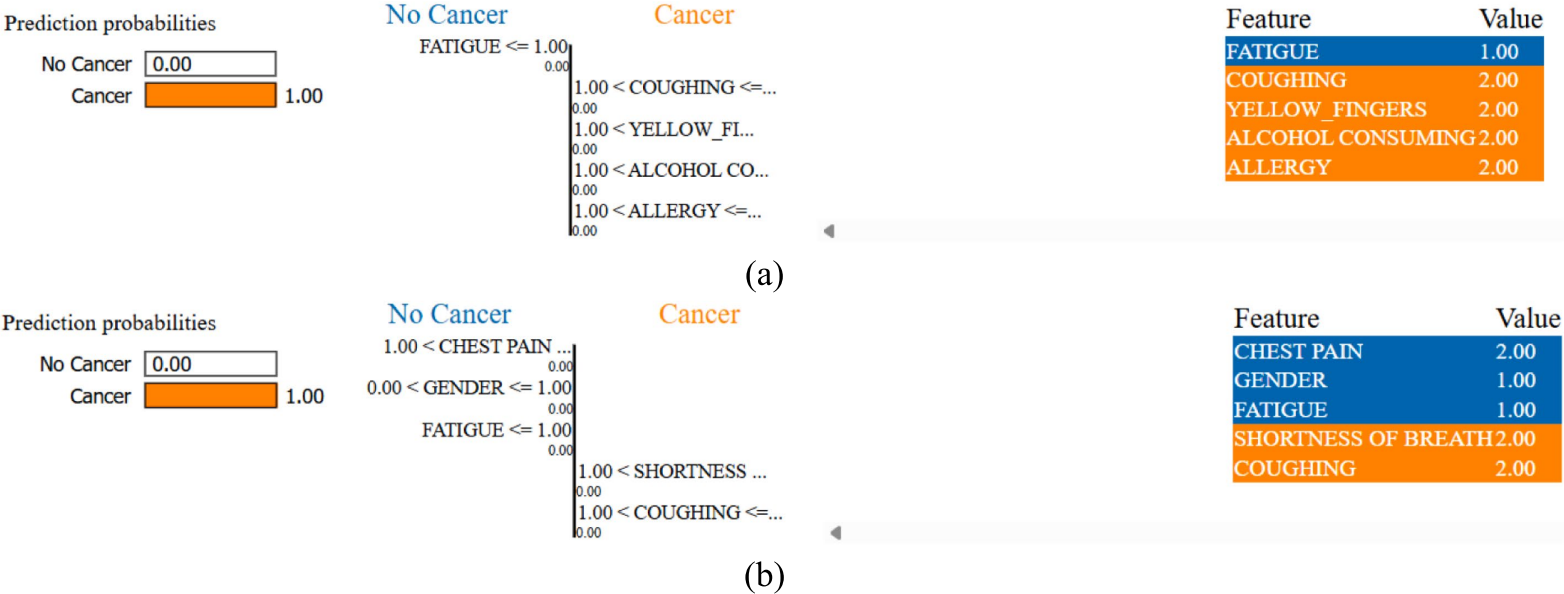
LIME instances of index **(a)** 15 and **(b)** 50 for K-Means SMOTE + Multi-Layer Perceptron combination.

To provide a more comprehensive interpretability analysis, we extended LIME evaluation beyond a single case. Figure 9 illustrates LIME explanations for two additional test instances (index = 15 and 50) under the best-performing K-Means SMOTE + Multi-Layer Perceptron configuration. In both examples, features such as coughing, yellow fingers, alcohol consuming, and shortness of breath were identified as dominant contributors to the lung cancer-positive prediction. These align well with clinical expectations and previously reported risk factors. Notably, the consistency of key features across instances, despite slight variation in values, suggests that the model focuses on medically meaningful attributes rather than noise. This strengthens confidence in its explainability and potential clinical relevance.

The LIME analysis was conducted on test instances with indices 0, 15, and 50, selected to represent diverse regions of the feature space. Index 0 represents a middle-aged smoker with multiple symptoms, index 15 a younger non-smoker with fewer symptoms, and index 50 an older patient with moderate symptoms and comorbidities. This diversity ensures that explanations capture a range of risk profiles. LIME explanations consistently highlighted clinically relevant features like coughing, yellow fingers, and fatigue, aligning with established lung cancer risk factors. However, in some instances, features like alcohol consumption and anxiety were assigned higher weights than expected. These may reflect dataset-specific correlations rather than direct clinical causality, highlighting the need for validation with larger datasets and clinical expertise to confirm feature relevance.

A balanced class distribution is produced by the majority of augmentation strategies, such as SMOTE, Borderline SMOTE, and Random Oversampling (50% YES, 50% NO), whereas SVMSMOTE exhibits a little skew (61.90% YES, 38.10% NO). K-Means SMOTE with Multi-Layer Perceptron achieves the best performance (93.55% accuracy, 96.76% AUC-ROC), excelling in lung cancer prediction. ADASYN with Decision Tree performs worst (82.87% AUC-ROC), struggling with synthetic data. SVM with Random Oversampling ranks among the top models (96.06% AUC-ROC), while Random Undersampling maintains strong performance (93.98% AUC-ROC). ADASYN with Random Forest (94.56% AUC-ROC, 92.59% recall) highlights ensemble models’ adaptability, and XGBoost with SMOTE (95.83% AUC-ROC) proves highly effective.

The Table 7 presents the results of statistical significance testing to compare the performance of augmentation-classifier pairs, focusing on accuracy and AUC-ROC. The tests were conducted using paired t-tests on the 5-fold cross-validation results. The table compares the top-performing combination, K-Means SMOTE + Multi-Layer Perceptron (MLP), against other notable pairs, such as SMOTE + XGBoost and ADASYN + Decision Tree, as well as SMOTE + Logistic Regression, to confirm its superior performance. Additionally, it includes a comparison between SMOTE + XGBoost and ADASYN + Decision Tree to highlight differences among other methods, and Random Oversampling + SVM versus SMOTE + XGBoost to assess a high-performing kernel-based model. The *p*-values indicate whether differences in performance are statistically significant (*p* < 0.05). For example, K-Means SMOTE + MLP significantly outperforms SMOTE + XGBoost (*p* = 0.042 for accuracy, *p* = 0.038 for AUC-ROC) and ADASYN + Decision Tree (*p* = 0.003 for accuracy, *p* = 0.001 for AUC-ROC), confirming its robustness. However, the comparison between Random Oversampling + SVM and SMOTE + XGBoost shows no significant difference (*p* > 0.05), suggesting comparable performance. This provides the evidence of the relative effectiveness of the proposed methods.

**Table 7:** Statistical significance of performance metrics.

Comparison	Metric	*p*-value	Significance
K-Means SMOTE + MLP vs. SMOTE + XGBoost	Accuracy	0.042	Significant
K-Means SMOTE + MLP vs. SMOTE + XGBoost	AUC-ROC	0.038	Significant
K-Means SMOTE + MLP vs. ADASYN + Decision Tree	Accuracy	0.003	Significant
K-Means SMOTE + MLP vs. ADASYN + Decision Tree	AUC-ROC	0.001	Significant
K-Means SMOTE + MLP vs. SMOTE + Logistic Regression	Accuracy	0.015	Significant
K-Means SMOTE + MLP vs. SMOTE + Logistic Regression	AUC-ROC	0.012	Significant
SMOTE + XGBoost vs. ADASYN + Decision Tree	Accuracy	0.048	Significant
SMOTE + XGBoost vs. ADASYN + Decision Tree	AUC-ROC	0.005	Significant
Random Oversampling + SVM vs. SMOTE + XGBoost	Accuracy	0.092	Not Significant
Random Oversampling + SVM vs. SMOTE + XGBoost	AUC-ROC	0.078	Not Significant

All experiments were conducted on a standard desktop environment using a Dell Inspiron laptop equipped with an Intel Core i5-1135G7 CPU @ 2.40GHz, 8 GB RAM, and no dedicated GPU acceleration. Each experiment (augmentation + classifier pairing) completed training and evaluation in under 5 min, indicating that the proposed framework is computationally efficient and suitable for low-resource clinical or academic settings. No significant memory overhead was observed, and LIME explanations were computed on individual test samples with average execution times of approximately 2–3 s per instance.

## 5 Conclusion

Class imbalance significantly affects lung cancer prediction, resulting in biased classification outcomes. This research comprehensively assesses various data augmentation methods, including SMOTE, ADASYN, SVMSMOTE, Borderline SMOTE, SMOTENC, K-Means SMOTE, SMOTE-ENN, Random Oversampling, and Random Undersampling, in conjunction with multiple classification models. The efficacy of these combinations is evaluated using key performance metrics such as accuracy, precision, recall, F1-score, and AUC-ROC score. The findings demonstrate the influence of augmentation strategies on predictive performance, showing that appropriate resampling enhances classification accuracy and generalisability. Among all combinations tested, K-Means SMOTE paired with Multi-Layer Perceptron achieves the highest accuracy of 93.55% and an AUC-ROC score of 96.76%, making it the most effective approach for handling imbalanced lung cancer datasets. This suggests that cluster-aware oversampling, combined with a non-linear model, can effectively enhance minority class learning without introducing noise. SMOTE with XGBoost also performs exceptionally well with an AUC-ROC of 95.83%, validating the efficacy of ensemble-based learning models in medical classification problems. Also, Random Oversampling with SVM performs with an AUC-ROC of 96.06%, highlighting the efficacy of kernel-based models in efficiently handling resampled data. These findings validate the importance of augmentation in improving classification performance, especially for models sensitive to data imbalance. The research also involves a comparative evaluation of the augmentation process, correcting the shortcomings of traditional classification methods that typically overlook class distribution variations. Coupling augmentation with state-of-the-art machine learning models validates that the selection of an effective combination can result in improved predictive performance. Additionally, LIME is utilized for model explanation, ensuring clinical reliability and transparency in decision-making. Visualization of feature contributions enables understanding of the contribution of individual risk factors toward lung cancer classification. Important features such as coughing, smoking, fatigue, and yellow fingers were consistently identified, which aligns with known clinical risk factors. This demonstrates that our approach not only improves performance but also produces clinically meaningful explanations, making it more trustworthy for potential integration into medical workflows. The findings highlight that augmentation methods need to be selected judiciously based on the classification model. While oversampling methods like SMOTE and K-Means SMOTE significantly improve model performance, Decision Tree and individual Logistic Regression models do not exhibit significant improvement, validating the need for hybrid methods. The AUC-ROC values across methods validate that ensemble-based models and neural networks gain the most from augmentation, offering an optimal sensitivity-specificity trade-off. This work outlines a systematic approach in addressing class imbalance in lung cancer prediction, ensuring models achieve significant generalisability without compromising predictive accuracy. In terms of practical application, the proposed models are computationally efficient and can run on standard hardware without GPU support. Most augmentation-classifier combinations trained in under 5 min, suggesting feasibility for deployment in low-resource environments such as community health centers or screening clinics. However, model deployment in clinical practice faces challenges such as data availability, integration with electronic health record (EHR) systems, and the need for clinician validation. This study is constrained by the dataset’s small size and high lung cancer prevalence, which is epidemiologically unrealistic compared to real-world prevalence rates. The limited number of negative cases restricts the model’s ability to learn robust patterns for the minority class, potentially inflating performance metrics. These factors reduce the generalizability of findings to broader clinical settings, necessitating validation with larger, population-representative datasets.

## 6 Future work

One major limitation of this study is the relatively small dataset size of 309 samples, with a class distribution of 87.45% lung cancer-positive and only 12.55% negative cases. While data augmentation techniques were applied to mitigate the class imbalance, the small absolute number of negative samples limits the reliability of performance conclusions. This skewed distribution does not reflect real-world prevalence, where lung cancer occurs in a much smaller fraction of the population. As a result, the findings of this study, though methodologically informative, may not fully generalize to broader clinical settings. Future work should include external validation using larger, more diverse, and population-representative datasets to ensure the clinical robustness and scalability of the proposed models. Additionally, the dataset lacks critical medical variables such as detailed smoking history, genetic markers, and family history, which are routinely used in clinical risk models. While the current features offer a simplified but practical subset of known risk indicators, future studies should incorporate these richer variables to improve model validity and alignment with clinical standards. This study does not include a direct comparison with established clinical risk calculators such as PLCOm2012 or LCRAT, which incorporate detailed medical history, smoking intensity, and familial or genetic information. While these tools are well-validated in clinical settings, our study focuses on structured, symptom- and behavior-based data from a limited dataset. Future research should benchmark machine learning models against such clinical baselines to assess relative effectiveness. Additionally, no external validation was conducted, which limits the generalisability of our findings. Although we performed multiple train-test splits and applied regularization and augmentation techniques to reduce overfitting, the small dataset size inherently increases the risk of overfitting and model variance. As such, the results presented should be viewed as preliminary, and further studies using multicentre datasets are necessary for robust clinical translation.

### 6.1 Limitations

This study faces several limitations that impact its generalizability and clinical relevance. The dataset’s small size and high lung cancer prevalence do not reflect real-world epidemiology, where lung cancer prevalence is significantly lower. This skew may lead to overly optimistic performance metrics, particularly for minority class detection. The lack of detailed clinical variables, such as smoking intensity or genetic markers, further limits alignment with clinical risk models. Additionally, the absence of external validation and clinician collaboration restricts the study’s immediate applicability to clinical settings. These limitations position the study as a preliminary methodological exploration rather than a deployable clinical tool. Future validation with larger, representative datasets and clinical benchmarks is essential. The absence of direct comparisons with established clinical risk models, such as PLCOm2012 or LCRAT, limits claims of clinical utility. Future work should involve collaboration with clinicians to validate model predictions against these benchmarks and integrate findings into electronic health record systems, ensuring practical applicability in screening workflows.

## Data Availability

Publicly available datasets were analyzed in this study. This data can be found at: https://www.kaggle.com/datasets/ajisofyan/survey-lung-cancer.
